# In Vitro Antithrombotic Properties of Salmon (*Salmo salar*) Phospholipids in a Novel Food-Grade Extract

**DOI:** 10.3390/md17010062

**Published:** 2019-01-18

**Authors:** Alexandros Tsoupras, Ronan Lordan, Katie Shiels, Sushanta Kumar Saha, Constantina Nasopoulou, Ioannis Zabetakis

**Affiliations:** 1 Department of Biological Sciences, University of Limerick, V94 T9PX Limerick, Ireland; Alexandros.Tsoupras@ul.ie (A.T.); Ronan.Lordan@ul.ie (R.L.); Ioannis.Zabetakis@ul.ie (I.Z.); 2 Shannon Applied Biotechnology Centre, Limerick Institute of Technology, Moylish Park, V94 E8YF Limerick, Ireland; Katie.Shiels@lit.ie (K.S.); Sushanta.Saha@lit.ie (S.K.S.); 3 Department of Food Science and Nutrition, School of the Environment, University of the Aegean, GR 81400 Myrina, Lemnos, Greece

**Keywords:** salmon, polar lipids, platelet aggregation, platelet-activating factor (PAF), thrombin, phosphatidylcholine, phosphatidylethanolamine, LC-MS, EPA, DHA, PUFA, phospholipids

## Abstract

Marine and salmon polar lipids (PLs) extracted by conventional extractions with non-food-grade solvents (CE-salmon-PLs) possess antithrombotic bioactivities against platelet-activating factor (PAF) and thrombin. Similar effects of food-grade-extracted (FGE) marine PLs have not yet been reported. In this study, food-grade solvents were used to extract PLs from Irish organic farmed salmon (*Salmo salar*) fillets (FGE-salmon-PLs), while their antithrombotic bioactivities were assessed in human platelets induced by platelet aggregation agonists (PAF/thrombin). FGE-salmon-PLs were further separated by thin layer chromatography (TLC) into lipid subclasses, and the antithrombotic bioactivities of each subclass were also assessed. LC-MS was utilized to elucidate the structure-activity relationships. FGE-salmon-PLs strongly inhibited PAF-induced platelet aggregation, while their relevant anti-thrombin effects were at least three times more potent than the previously reported activities of CE-salmon-PLs. TLC-derived lipid fractions corresponding to phosphatidylcholines (PC) and phosphatidylethanolamines (PE) were the most bioactive lipid subclasses obtained, especially against thrombin. Their LC-MS analysis elucidated that they are diacyl- or alkyl-acyl- PC and PE moieties baring ω3 polyunsaturated fatty acids (PUFA) at their *sn*-2 position, such as eicosapentaenoic acid (EPA) or docosahexaenoic acid (DHA). Our results concerning the potent antithrombotic effects of FGE-salmon-PLs against both PAF and thrombin pathways strongly suggest that such food-grade extracts are putative candidates for the development of novel cardioprotective supplements and nutraceuticals.

## 1. Introduction

Marine polar lipids (PLs) have exhibited several beneficial activities against inflammation, thrombosis, and related disorders [1,2,3,4]. In contrast to triglycerides and esters, marine PLs have superior incorporation into cell membranes and plasma lipoproteins, including high-density lipoprotein (HDL) [1,2,3,4]. Therefore, they not only possess higher bioavailability of their ω3 polyunsaturated fatty acids (PUFA), but they can more efficiently affect the biofunctionality of plasma lipoproteins and the activities of several plasma membrane receptors related to important pathways of inflammation and thrombosis [1,2,3,4,5], such as the receptor for platelet-activating factor (PAF) [4,5,6].

PAF, namely 1-*O*-alkyl-2-acetyl-*sn*-glycero-3-phosphocholine [6], is a unique physiologically active signalling phospholipid activator of many cell types, including platelets, and a potent mediator of inflammation and related diseases, such as atherosclerosis and cardiovascular diseases (CVD), renal disorders, cancer, persistent infections, etc. [5]. Another important mediator of platelet pathophysiology and atherothrombosis is thrombin [7,8]. Thrombin is a serine protease that participates in coagulant catalytic processes, such as the conversion of fibrinogen into fibrin and the activation of the V, VIII, XI, and XIII coagulation factors [7,8], as well as the activation of many cell types and platelets including platelet aggregation [7]. Such soluble platelet agonists are produced during coagulation (e.g., thrombin) and inflammation (e.g., PAF) and play a critical role via G-protein-coupled receptors (GPCR) in platelet activation and thrombus formation [7]. These mediators also present in high concentrations in atherosclerotic plaques and atherothrombotic events [6,8].

Previously, these two pathways were considered independent, and therapeutic approaches were mainly focused on each individual pathway. However, there is a crosstalk between inflammation and coagulation systems in chronic diseases, whereby inflammation leads to the activation of coagulation, and coagulation also considerably affects inflammatory activity [6]. For example, PAF has been found to synergistically augment thrombin-induced platelet activation [9], while in melanoma metastasis the PAF/PAF-receptor pathway interrelates with pathways related to thrombin and its receptor (PAR-1) [10]. Vice versa, thrombin stimulation of endothelial cells through its receptor PAR-1 results in the activation of calcium-independent phospholipase A_2_ (iPLA_2_). Thus, the synthesis of membrane phospholipid-derived inflammatory mediators such as PAF, arachidonic acid (AA), and prostaglandins [11] are all considered to be central in both the initiation and propagation of the inflammatory response [6]. Moreover, PAF produced by endothelial cells treated with thrombin can further stimulate and influence the activation of circulating effector cells, such as platelets and polymorphonuclear leukocytes [12], while the interaction of the endothelium with activated circulating blood cells takes place in both physiologic conditions and in syndromes of vascular injury [6,12].

As a result, the study of bioactive molecules and compounds, especially of natural origin, with both anti-PAF and anti-thrombin activities is of significant importance. Marine PLs have demonstrated strong anti-inflammatory and antithrombotic activities by inhibiting the PAF/PAF-receptor-related pathways and thus PAF activities [4,5,13,14,15,16], but also by modulating its metabolism towards homeostatic PAF levels [17,18]. The inhibitory effects of marine PLs against PAF may be directly related to their effect on PAF-receptor or indirectly by their lipid rafts that are able to influence the PAF-receptor lipid-membrane microenvironment, or both cases may happen simultaneously, which seem to be the most probable scenario, thus explaining their synergistic effect [4,5,16].

Through these mechanistic effects, marine PLs have exhibited in vitro and in vivo beneficial anti-atherogenic and cardioprotective properties [4,5,13,14,15,16,17,18,19]. It has recently been demonstrated that PLs extracted from fillets of Irish organic farmed salmon (*Salmo salar*), also possess strong in vitro antithrombotic bioactivities against platelet aggregation, mostly through their strong inhibitory effects against the PAF pathway, but with less potent effects towards the thrombin pathway [16]. These salmon PL extracts were previously extracted from salmon fillets, by utilising conventional extraction methods (CE) based on non-food-grade solvents [16,20,21].

However, to the best of our knowledge, there is no reported evidence for similar antithrombotic bioactivities of marine PLs extracted from fish species using food-grade solvents, according to European Union (EU) legislation for edible fish oil extractions. For the first time, the in vitro antithrombotic effects of salmon PL extracts that were extracted from Irish organic farmed salmon (*Salmo salar*) fillets using food-grade solvents (FGE-salmon-PLs), against both the PAF and thrombin pathways of platelet aggregation are presented. FGE-salmon-PLs were further separated into several lipid subclasses and the biological activity of each subclass was assessed against PAF and thrombin-induced platelet aggregation. LC-MS analysis was conducted on the most bioactive lipid subclasses in order to elucidate the structure–activity relationships.

The present study is a continuation of previous research towards the development of novel PL-based cardioprotective food supplements and nutraceuticals from sustainable marine sources.

## 2. Results

### 2.1. Yield of FGE-salmon-PLs

Total lipids (TL) of all samples (*n* = 6) of salmon fillets from the same batch of farmed salmon were extracted using food-grade solvents according to EU legislation for extracting fish oils and further separated into neutral lipids (NL) and PLs by a counter-current distribution, also using food-grade solvents. The amounts of FGE-salmon-PLs obtained (expressed as g of lipids per 100 g of fish tissue) are given in Table 1. They were found to be similar (non-statistically significant difference: *p* > 0.05) with that of previously reported outcomes for salmon PLs extracted by conventional extractions (CE-salmon-PLs) using non-food-grade solvents, such as chloroform and petroleum ether [16].

### 2.2. TLC Analysis of FGE-Salmon-PLs

**Figure 1:**
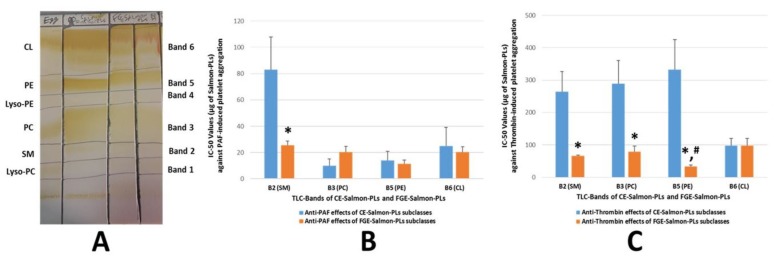
TLC analysis of both food-grade-extracted (FGE)-salmon-polar lipids (PLs) and conventional extractions with non-food-grade solvents (CE)-salmon-PLs (**A**), and inhibitory effects (IC_50_ values) of lipid fractions (TLC bands) against platelet aggregation induced by platelet-activating factor (PAF) (**B**) or thrombin (**C**). (**A**): 1st column (from left to right): Separation of a standard mixture of egg-yolk phospholipids, 2nd column: Separation of CE-salmon-PLs, 3rd and 4th columns: Separation of FGE-salmon-PLs. (**B** and **C**): Results are expressed as mean values of IC_50_ against PAF/thrombin-induced platelet aggregation; the blue bars depict the IC_50_ values of each TLC band of the CE-salmon-PL extracts, while the orange bars depict the IC_50_ values of each TLC band of the FGE salmon-PL extracts. The IC_50_ values of TLC bands of the CE-salmon-PLs against the PAF-induced aggregation of human platelet-rich plasma (hPRP) are reproduced in this figure (blue bars in **B**) reproduced with permission according to Tsoupras et al. [16]. * indicates statistically significant differences (*p* < 0.05) between the bioactivity of FGE-salmon-PL fractions in comparison to that of CE-salmon-PL fractions. # indicates statistically significant differences (*p* < 0.05) between the bioactivity of FGE-salmon-PE fraction against thrombin in comparison to the relative bioactivity of all the other FGE-salmon-PLs fractions. Lipid fractions of TLC bands 1 and 4 (corresponding to lyso-phosphatidylcholines (PC) and Lyso-phosphatidylethanolamines (PE) did not exhibit inhibitory bioactivities. The results are representative of six independent experiments, in order to ensure reproducibility.

FGE-salmon-PLs were further separated into several PL subclasses and fractions by preparative TLC analysis, as previously described [16]. It was found that within the FGE-salmon-PLs, several phospholipid subclasses exist determined by the TLC bands when compared to specific standards of phospholipid subclasses, as shown in Figure 1A. TLC bands 1–6 of the FGE-salmon-PLs were found to possess similar R_f_ values to those of lyso-phosphatidylcholines (L-PC), polar lipids of the sphingomyelin family (SM), phosphatidylcholines (PC), lyso-phosphatidylethanolamines (L-PE), phosphatidylethanolamines (PE), and cardiolipin (CL) respectively, see Figure 1A. These results are in accordance with previously reported results of similar TLC analyses of the CE-salmon-PLs and CE-marine-PLs derived from several other fish species [15,16].

### 2.3. Antithrombotic Effects of FGE-Salmon-PLs and Their Lipid Subclasses against Human Platelet Aggregation

The in vitro antithrombotic effects of FGE-salmon-PLs against aggregation of human platelets was evaluated by the IC_50_ values of their inhibitory effects towards platelet aggregation induced by well-established aggregation agonists such as PAF and thrombin in human platelet-rich plasma (hPRP), as previously described [16]. The IC_50_ values reflect the inhibitory strength of each salmon PL extract, because low IC_50_ values indicate stronger inhibition of PAF-induced/thrombin-induced platelet aggregation for a given salmon PLs’ concentration. The mean IC_50_ value of the inhibitory effect of all the FGE-salmon-PLs extracts against PAF-induced platelet aggregation was found to be within the same range (non-statistically significant difference: *p* > 0.05) with their relative IC_50_ value towards the thrombin-induced platelet aggregation, see Table 1. Both of these IC_50_ values are comparable with relative IC_50_ values of bioactive PLs extracted from other fish species, but also from other food samples and microorganisms, which exhibited similar anti-inflammatory and antithrombotic effects towards PAF-induced activation and aggregation of platelets [13,14,15,22,23,24].

With respect to salmon, the FGE-salmon-PLs extracts exhibited lower inhibitory effects against the PAF pathway of human platelet aggregation in comparison with the previously reported anti-PAF effects of the CE-salmon-PLs extracts [16]. However, the IC_50_ values of the FGE-salmon-PLs against PAF-induced hPRP aggregation were in the same order of magnitude as that of the CE salmon-PLs. On the other hand, FGE-salmon-PLs exhibited much higher inhibitory effects towards the thrombin-pathway of human platelet aggregation than the previously reported anti-thrombin effects of the CE-salmon-PLs (*p* < 0.05) [16]. The IC_50_ values of FGE-salmon-PLs against thrombin-induced hPRP aggregation were also comparable with relative IC_50_ values of bioactive CE-PLs extracts from other fish products (Greek avgotaracho) and cyanobacteria, which exhibited similar effects towards thrombin-induced aggregation of platelets [22,23].

Furthermore, the PLs subclasses in each of the TLC bands, which were obtained by the TLC separation of the FGE-salmon-PLs, were also tested for their ability to inhibit platelet aggregation of hPRP induced by PAF and thrombin, see Figure 1. The TLC separation of both FGE-salmon-PLs and CE-salmon-PLs in TLC bands (TLC-derived lipid fractions corresponding to specific lipid subclasses) is depicted in Figure 1A, while the IC_50_ values of the bioactive lipid fractions of each TLC band are given in Figure 1B,C. The FGE-salmon-PLs extract exhibited inhibitory properties, that are attributed to almost all the polar lipid fractions (Bands 2, 3, 5, 6) apart from lipid fractions 1 and 4 that did not exhibit such an effect, see Figure 1B,C. None of the fractions exhibited any aggregatory properties of hPRP.

Similarly to previously reported results of the CE-salmon-PLs [19], the TLC bands 3 and 5 of the FGE-salmon-PLs, which have similar R_f_ values to those of PC and PE lipid subclasses, see Figure 1A, exhibited potent inhibitory effects towards PAF-induced aggregation of hPRP, see Figure 1B. These results are in accordance with previously reported outcomes against PAF for these PL subclasses (PC and PE) present in the same TLC bands of PLs derived from salmon and several other fish species [14,15]. However, TLC band 2 of the FGE-salmon-PLs, which has a similar R_f_ value to that of the sphingomyelin (SM) family band, also exhibited potent inhibitory effects towards PAF-induced aggregation of hPRP, see Figure 1B, which was much higher than the previously reported relative one of the CE-salmon-PLs [16] (*p* < 0.01). TLC band 6 of the FGE-salmon-PLs exhibited a similar anti-PAF effect with the previously reported relative activity of the CE-salmon-PLs [16], see Figure 1B. 

Moreover, the TLC bands 2, 3, and 5 of the FGE-salmon-PLs, which have similar R_f_ values to those of the SM family, PC, and PE lipid subclasses, exhibited much higher inhibitory effects towards thrombin-induced platelet aggregation of hPRP than the relative activity of the CE-salmon-PLs (*p* < 0.01 in all these comparisons) (Figure 1C). Furthermore, the fraction of the SM family of the FGE-salmon-PLs exhibited higher anti-PAF and anti-thrombin activities in comparison to the reported anti-PAF and anti-thrombin activities of the fraction of the SM family of the CE-salmon-PLs [16]. However, the anti-thrombin effects of the fraction of the SM family of the FGE-salmon-PLs was found to be similar to the relative anti-thrombin effects of the PC fraction (*p* > 0.05) and significantly lower to that of the PE fraction (*p* < 0.05) of the FGE-salmon-PLs. In addition, the anti-PAF effects of the fraction of the SM family of the FGE-salmon-PLs were found to be slightly lower (but not significantly lower, *p* > 0.05) than the relative effect of the PC fraction, whereas it was significantly lower (*p* < 0.05) than the relative effect of the PE fraction of the FGE-salmon-PLs. It should be mentioned that TLC band 5 of the FGE-salmon-PLs (that corresponds to the PE subclass) exhibited the highest inhibitory effect towards thrombin-induced aggregation of hPRP, when compared with the relative activities of either all the other TLC bands of the FGE-salmon-PLs (*p* < 0.05) or all the TLC bands of the CE-salmon-PLs (*p* < 0.01).

### 2.4. LC-MS Analysis of FGE-salmon-PLs and Structure–Activity Relationships

**Figure 2:**
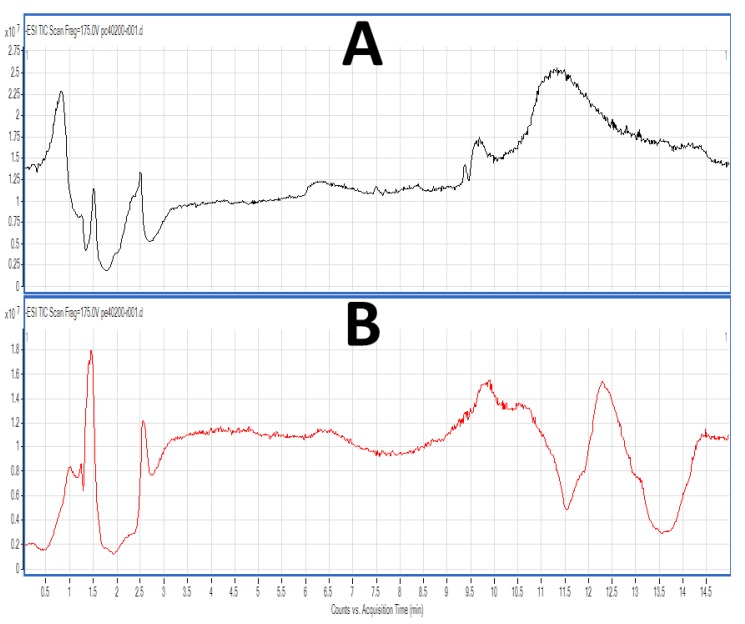
Representative HPLC chromatograms of the most bioactive lipid subclasses of the FGE-salmon-PLs. (**A**) Depicts a representative chromatogram of the TLC fraction corresponding to the phosphatidylcholines (PC) lipid subclass of the FGE-salmon-PLs, whereas (**B**) depicts a representative chromatogram of the TLC fraction corresponding to the phosphatidylethanolamines (PE) lipid subclass of the FGE-salmon-PLs.

FGE-salmon-PLs and the most bioactive TLC fractions (corresponding to the PC and PE lipid subclasses), were further analysed by LC-MS as previously described [16]. Characteristic chromatograms of the HPLC separation for both TLC fractions of PC and PE lipid subclasses of the FGE-salmon-PLs are depicted in Figure 2A,B respectively. Several peaks with specific retention times were observed in both cases. By using a C18 reverse phase column in the LC-MS analysis, the separation of the lipids is mostly based on the length of the non-polar acyl- or alkyl-groups in combination with their degree of unsaturation. Thus, PC and PE species baring PUFA were separated within short retention times (1–3.5 min), rather than other PC and PE species baring more saturated and longer chains within their structures that seem to be eluted in higher retention times (8–15 min).

By applying quadrupole time-of-flight mass spectrometry (Q-TOF) simultaneously with the HPLC separation for each one of these peaks, unique MS data were obtained for each peak leading to complete structural elucidations of novel structures for these PC and PE moieties. The characterisation of these molecules was based on the acquired *m/z* values of the dehydrogenated negative ions [M − H]^−^ for PE and free fatty acids (FFA), and the demethylated negative ions [M − CH_3_]^−^ for PC as previously described [16], and further verified by using the LIPID MAPS: Nature Lipidomics Gateway (www.lipidmaps.org), based on the lowest delta values during identification, in combination with their fatty acids contents that were acquired by the LC-MS analyses of the FFA derived by the saponification of FGE-salmon-PLs.

**Figure 3:**
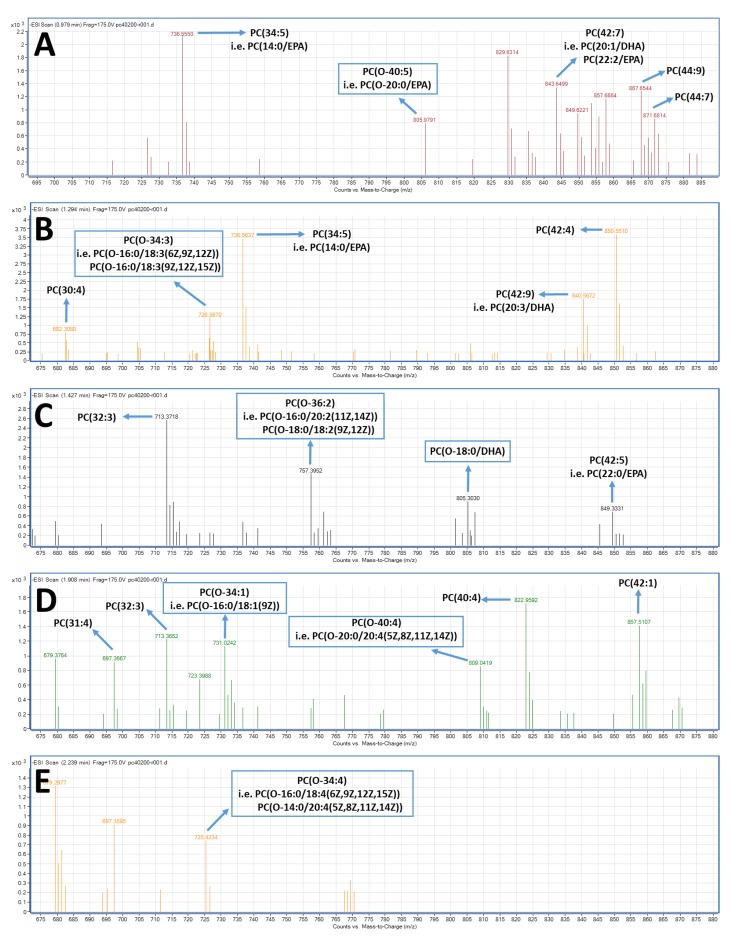
Representative mass spectra of PC species present in the relative TLC fraction corresponding to the PC lipid subclass of the FGE-salmon-PLs eluted over short retention times (1.0–3.5 min).

**Figure 4:**
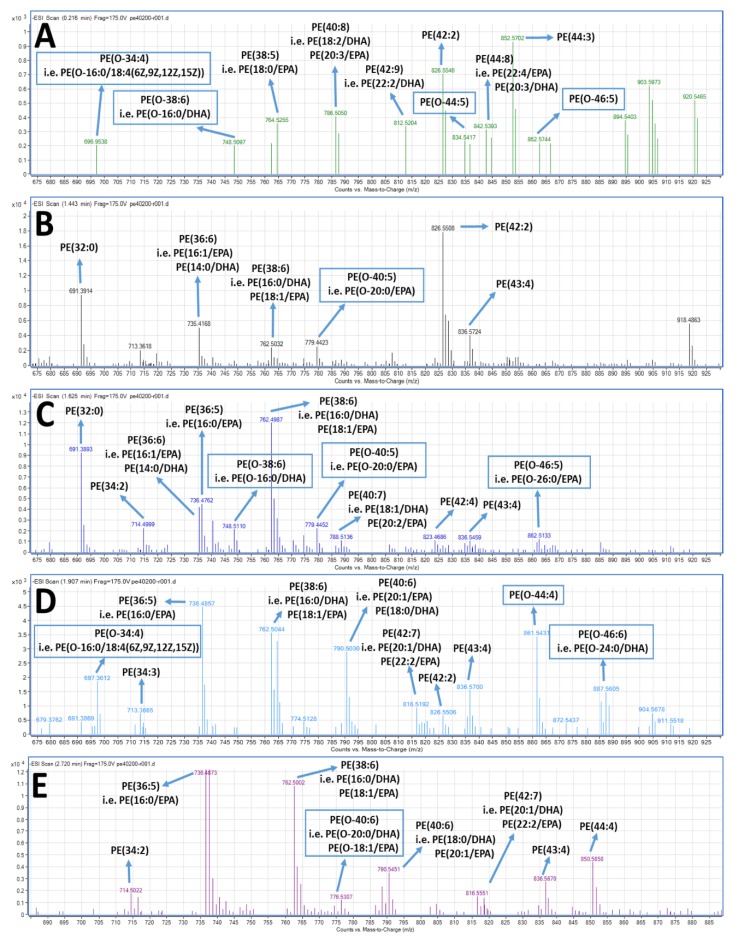
Representative mass spectra of PE species present in the relative TLC fraction corresponding to the PE lipid subclass of the FGE-salmon-PLs eluted in short retention times (1.0–3.5 min).

With respect to the LC-MS structural analysis of the PC and PE fractions, survey scans in the negative ion mode between 600 and 1000 *m/z* of MS1 demonstrated a specific pattern of molecular species for PC, see Figure 3, and PE, see Figure 4, eluted within short retention times (1–3.5 min). It is proposed that many of these are diacyl-PC and diacyl-PE species containing 14:0, 16:0, 16:1, 18:0, 18:1, 18:2, 20:0, 20:1, 20:2, 20:3, 22:0, 22:1, 22:2, etc., fatty acids at the *sn*-1 position and mostly ω3 PUFA at the *sn*-2 position, such as the 22:6 fatty acid (DHA) or the 20:5 fatty acid (EPA). 1-*O*-alkyl-2-*sn*-acyl-PC moieties also seem to be present in less but considerable quantities, usually with ω3 PUFA at the sn-2 position, including DHA and EPA. shown as squares in Figure 3 and Figure 4, respectively. The most representative novel mass spectra of these acyl-acyl PC and PE moieties, but also of the alkyl-acyl PC and PE moieties are shown in Figure 3 and Figure 4, respectively.

In detail, specific alkyl-acyl-PC molecules baring either DHA or EPA at the *sn*-2 position seem to be present in the TLC fraction of the bioactive PC lipid subclass of the FGE-salmon-PLs, such as the 1-*O*-alkyl-(20:0)-2-*sn*-alkyl-(20:5,EPA)-3-PC, as shown in Figure 3A, with a theoretical mass of 821.63 (with a relative demethylated negative ion [M−CH_3_]^−^ at 805.98 *m/z*) and the 1-*O*-alkyl-(18:0)-2-*sn*-alkyl-(22:6,DHA)-3-PC, see Figure 3B, with a theoretical mass of 819.61 (with a relative demethylated negative ion [M−CH_3_]^−^ at 805.30 *m/z*). Furthermore, specific alkyl-acyl-PE molecules baring either DHA or EPA at the *sn*-2 position seem also to be present in the TLC fraction of the bioactive PE lipid subclass of the FGE-salmon-PLs, such as the 1-*O*-alkyl-(16:0)-2-*sn*-alkyl-(22:6,DHA)-3-PE, see Figure 4C, with a theoretical mass of 749.54 (with a relative dehydrogenated negative ion [M − H]^−^ at 748.51 *m/z*), the 1-*O*-alkyl-(20:0)-2-*sn*-alkyl-(20:5,EPA)-3-PE, see Figure 4C, with a theoretical mass of 779.58 (with a relative dehydrogenated negative ion [M − H]^−^ at 779.44 *m/z*), the 1-*O*-alkyl-(24:0)-2-*sn*-alkyl-(22:6,DHA)-3-PE, see Figure 4D, with a theoretical mass of 887.73 (with a relative dehydrogenated negative ion [M − H]^−^ at 887.56 *m/z*), and the 1-*O*-alkyl-(20:0)-2-*sn*-alkyl-(22:6,DHA)-3-PE, see Figure 4E, with a theoretical mass of 791.55 (with a relative dehydrogenated negative ion [M − H]^−^ at 790.55 *m/z*), or the 1-*O*-alkyl-(18:1)-2-*sn*-alkyl-(20:5,EPA)-3-PE, see Figure 4E, with the same theoretical mass of 791.55 (with a same relative dehydrogenated negative ion [M − H]^−^ at 790.55 *m/z*). 

Notably, PC and PE species eluted in higher retention times (8–15 min), seem to have longer and more saturated carbon chains of their acyl-moieties. MS data of these diacyl-PC and diacyl-PE species (with longer and more saturated fatty chains at both the *sn*-1 and *sn*-2 positions) are not shown, since such PC and PE species do not seem to possess any effect against platelet aggregation and have not been previously reported to possess such bioactivity.

**Figure 5:**
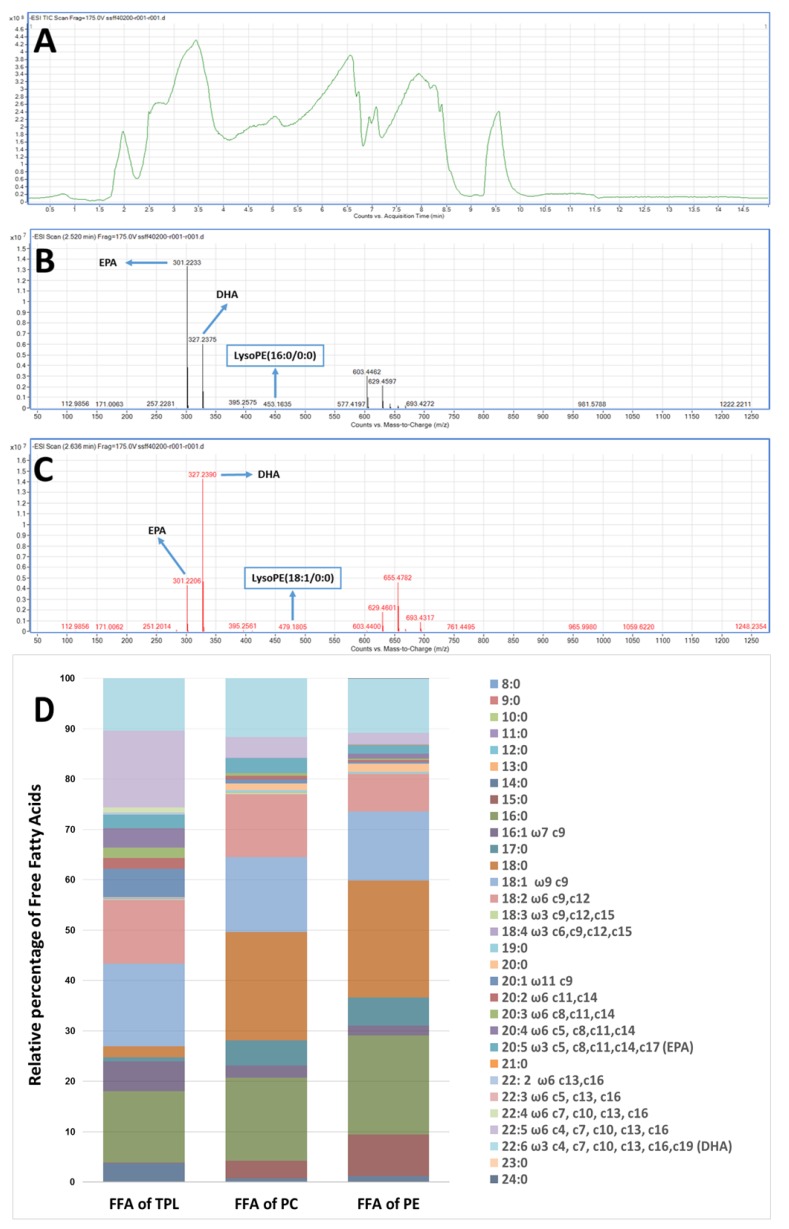
Representative chromatogram and mass spectra of the most abundant ω3 PUFA and free fatty acids (FFA) composition of the FGE-salmon-PLs, PC, and PE fractions. (**A**) Depicts a representative chromatogram of the LC-MS analysis of the FFA derived by the saponification of the FGE-salmon-PLs. (**B**,**C**) depict representative mass spectra of the most abundant ω3 PUFA (EPA and DHA), while some characteristic lyso-PL moieties that were detected are also presented in squares. (**D**) Depicts the FFA composition of the FGE-salmon-PLs, PC, and PE fractions acquired by LC-MS analysis after saponification of these samples.

Furthermore, the data of the LC-MS-analysis of the FFA composition derived from the saponification of FGE-salmon-PLs and of both PC and PE fractions are shown in Figure 5. Figure 5A shows a characteristic chromatogram of the LC-MS analysis of the FFA derived by the saponification of the FGE-salmon-PLs. Survey scans performed in the negative ion mode between 200 and 1000 *m/z* of MS1 showed that in all FGE-salmon-PLs samples, PUFA were the most abundant fatty acid class (approximately 50% of the total FFA) followed by saturated fatty acids (SFA, approximately 21% of the total FFA) and monounsaturated fatty acids (MUFA, approximately 14% of the total FFA). More specifically, salmon TPL contains high amounts of ω3 PUFA, with the most abundant being the EPA (20:5ω3) and the DHA (22:6ω3), see Figure 5B–D. Specific Lyso-PC and Lyso-PE moieties were also detected, such as Lyso-PE (16:0/0:0), Lyso-PE (18:1/0:0), Lyso-PE (20:0/0:0), Lyso-PC (O-18:0/0:0), etc., see Figure 5B,C. These data indicate that ω3 PUFA (EPA and DHA) were released during the saponification of FGE-salmon-PLs mostly from their *sn*-2 position. In addition, the most abundant ω6 fatty acids in the FGE-salmon-PLs samples were docosapentaenoic acid (DPA; 22:5ω6), AA (20:4ω6), and linoleic acid (LA; 18:2ω6), while the most abundant MUFA was oleic acid (18:1 c9) and the most abundant SFA were palmitic acid (16:0) and stearic acid (18:0), see Figure 5D. Τhe relative ratio of ω6/ω3 was found to be approximately 2.8/1. 

Similarly, the most abundant fatty acids found in the FFA derived from the PC and PE fractions were the ω3 fatty acids EPA (20:5ω3) and DHA (22:6ω3) and the ω6 fatty acids DPA (22:5ω6) and linoleic acid (LA; 18:2ω6) from the PUFA, the palmitic acid (16:0) and stearic acid (18:0) from the SFA, and the oleic acid (18:1 c9) and the palmitoleic acid (16:1 c9) from the MUFA. In addition, the PC fraction was not found to contain AA (20:4ω6), while the PE fraction contained much less AA than the FGE-salmon-PLs, implying that the AA contained in the FGE-salmon-PLs is distributed in PLs subclasses other than PC and PE. Interestingly, both PC and PE fractions were found to be rich in odd-chain SFA such as the pentadecanoic acid (15:0) and heptadecanoic acid (17:0). The ω3 fatty acid content of both the PC and PE fractions were similar to that of their content in ω6 fatty acids, and thus the relative ratios of ω6/ω3 for both fractions were found to be approximately 1.1 and 0.9 respectively, which were lower than the relative ratio found in the FGE-salmon-PLs extract.

**Figure 6:**
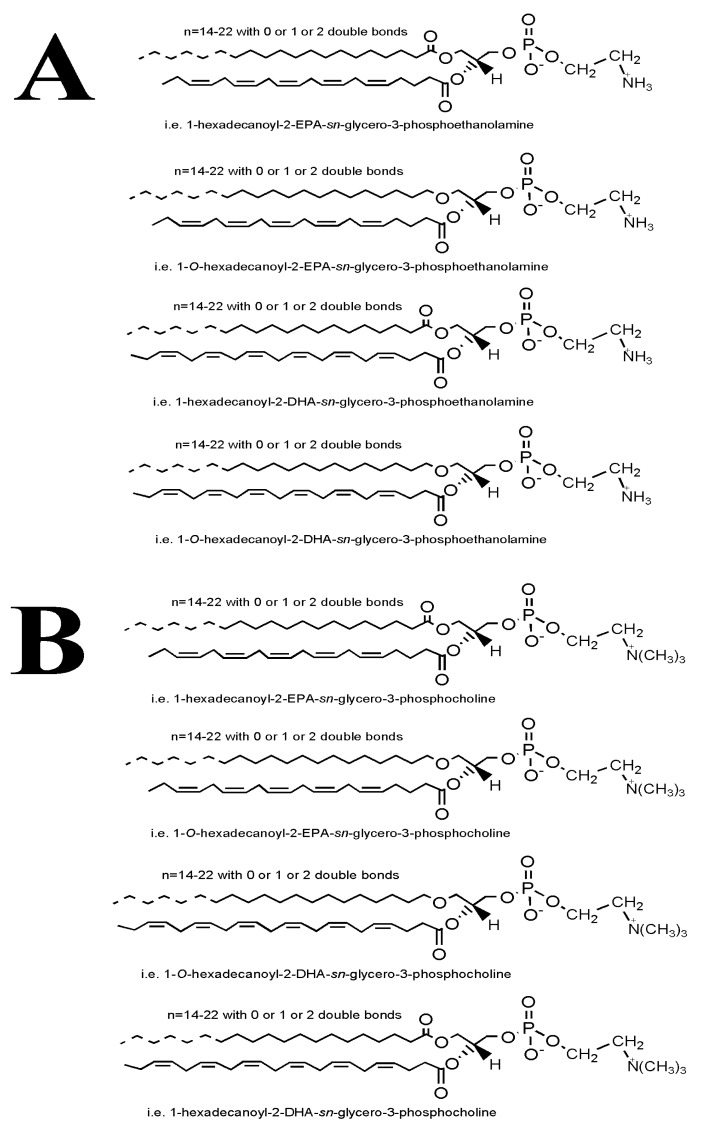
Proposed structures of (**A**) 1-acyl-2-ω3PUFA-PE/1-alkyl-2-ω3PUFA-PE and (**B**) 1-acyl-2-ω3PUFA-PC/1-alkyl-2-ω3PUFA-PC moieties, which according to the LC-MS analysis were found to be present in the bioactive PE and PC fractions of the FGE-salmon-PLs.

Overall, our results indicate that the most important acyl-acyl-PC and acyl-acyl-PE moieties, but also those of the alkyl-acyl PC and alkyl-acyl PE moieties, are those that bare ω3 PUFA (i.e., EPA and DHA) in their *sn*-2 position of the glycerol backbone. Representative proposed structures of these molecules are shown in Figure 6.

## 3. Discussion

Inflammatory and thrombotic events are implicated in all stages of atherosclerosis and CVD [5]. Apart from endothelial cells and leukocytes, activation and aggregation of platelets is also a key player to the “crosstalk” that takes place between various cells during vascular physiology and pathophysiology, and thus during several inflammation-related atherosclerotic and atherothrombotic events of cardiovascular diseases [5,25]. Several platelet agonists can induce platelet activation and aggregation, with PAF and thrombin being the most potent agonists [5,6,7,8,25].

As a result, the study of bioactive molecules and compounds, especially of natural origin, with both anti-PAF and anti-thrombin activities is of potential therapeutic value. Indeed, the consumption of fish oils has improved platelet function, human thrombosis, and haemostasis in several disorders [26,27,28]. This has primarily been attributed to EPA and DHA through mechanisms related to the eicosanoid pathways. For example, EPA can replace AA in platelet membrane phospholipids and acts as a substrate for cyclo-oxygenase [29], whilst DHA, but not EPA, can reduce collagen-induced platelet aggregation, either through replacement of AA by DHA in platelet phospholipids, through the inhibition of cyclooxygenase, or through the direct effects of DHA on platelet function independent of eicosanoid production. [30,31,32]. Platelets treated with EPA and DHA can modify platelet membranes and reduce the rate of thrombin generation, which results in reduced platelet procoagulant activity and thrombus formation [33]. Dietary fish oil supplementation rich in EPA and DHA also reduces collagen or thrombin-induced platelet aggregation, TXB_2_ generation from platelets and platelet membrane fluidity in normolipemic subjects [34]. Dietary supplementation of fish oil (MaxEPA) rich in EPA and DHA for six weeks resulted in the reduction of PAF synthesis in healthy subjects, while PAF generation was also reduced in monocyte monolayers in the EPA treatment group only, while PAF synthesis was increased by AA [35].

On the other hand, administration of these ω3 PUFA (either EPA or DHA) in the form of ethyl esters was not able to reduce PAF-induced platelet aggregation or affect fibrinolytic and vascular function [31]. In addition, several recent reviews and meta-analyses have indicated that there is insufficient evidence for any benefits from ω3 PUFA supplements, in the form of purified fatty acids or in the form of esters, on reducing the risk for CVD (either on primary or in secondary prevention) and on lowering the risk of all-cause mortality, cardiac death, sudden death, myocardial infarction, stroke, or cancer [36,37,38].

Remarkably, several of these studies have also proposed that the beneficial effect of fish intake on cerebrovascular risk is likely to be mediated through the interplay of a wide range of nutrients abundant in fish [4]. Indeed, promising results have been attributed to several PLs of marine origin [4,5,13,14,15,16,18,19,22,23], and of natural origin in general [5,24]. Marine PLs and especially, those baring ω3 PUFA, possess a plethora of beneficial bioactivities towards inflammation-related disorders [1,2,3,4], including their far superior incorporation into cell membranes and plasma lipoproteins such as high-density lipoprotein (HDL), compared to the incorporation of marine triglycerides or esters, and thus they possess higher bioavailability of their bioactive ω3 PUFA in several tissues, including those with difficult accessibility (i.e., the brain tissue because of the blood–brain barrier) [4,39,40]. 

Marine PLs possess strong anti-inflammatory and antithrombotic activities against PAF-related pathways and metabolism [4,5,13,14,15,16,17,18] and against the thrombin pathways [16,22]. However, in these previous studies, the bioactive polar lipids were extracted using well-established but conventional methods (CE-marine-PLs), such as the Bligh and Dyer [20] method and the Galanos and Kapoulas [21] counter-current distribution, which are based on solvents such as chloroform, methanol, and petroleum ether. Although these well-established methods are reliable for acquiring a high yield of polar lipids from various food sources, they are not the panacea for extracting and separating bioactive polar lipids.

To the best of our knowledge, anti-PAF and anti-thrombin activities of polar lipids derived from marine sources by environmentally clean food-grade extraction methods according to EU legislation without the use of organic toxic solvents such as chloroform and petroleum ether, have not previously been reported. However, there have been several studies that have evaluated the effect of using food-grade and non-food-grade extraction methods on the fatty acid composition of lipid extracts of marine origin, especially in phospholipids rich in PUFA such as EPA and DHA [41,42,43]. However, these studies did not focus on the bioactivities that such marine-derived PLs may possess. This study is the first to report that polar lipids derived from salmon using food-grade extraction methods compliant with current EU legislation (Directive 2009/32/EC) possess strong antithrombotic activities against both PAF and thrombin-induced human platelet aggregation.

Furthermore, in our study, it was also found that the anti-PAF effects of the FGE-salmon-PLs were within the same order of magnitude as those previously reported from CE-salmon-PLs [16], while the anti-thrombin effects of the FGE-salmon-PLs were at least three times more potent than the previously reported relevant effects of the CE-salmon-PLs.

In addition, specific TLC lipid fractions of the FGE-salmon-PLs corresponding to PC and PE lipid subclasses exhibited the most potent inhibitory effect against PAF-induced human platelet aggregation, in comparison to all of the other TLC lipid fractions. These results are in accordance with previously reported activities against PAF for PC and PE PLs present in the same TLC bands of PLs derived from CE-salmon-PLs and several other fish species [14,16].

Moreover, for the first time, TLC-derived lipid fractions corresponding to PC and PE lipid subclasses of the FGE-salmon-PLs exhibited much higher inhibitory effects against the thrombin-induced human platelet aggregation compared to the anti-thrombin effects of the relative TLC-derived lipid fractions of PC and PE of the CE-salmon-PLs. These effects can be attributed to the existence of potent anti-thrombin PC and PE lipid moieties within the FGE-salmon-PLs that are not present within the CE-salmon-PLs. In order to clarify these differences, structural elucidation of the bioactive PC and PE lipid fractions of the FGE-salmon-PLs was conducted by LC-MS analysis. 

The overall fatty acid profile of the FGE-salmon-PLs and the bioactive PC and PE fraction in this study have similarities with previously reported salmon lipid profiles [16], but they are also similar to phospholipids of several other fish and animals [44,45,46,47,48]. Similarly to the obtained results in the CE-salmon-PLs and in other relative studies in salmon [16,44,45], the FGE-salmon-PLs from Irish organic farmed salmon were found to contain high levels of ω3 PUFA, with the most abundant ω3 fatty acids being EPA and DHA. However, concerning the observed differences, the PE fraction of FGE-salmon-PLs was found to be abundant in both EPA and DHA ω3 PUFA, in contrast to the PE fraction of the CE-salmon-PLs. In addition, in contrast to the CE-salmon-PLs, the ω6-PUFA AA was not present in the PC fraction of the FGE-salmon-PLs, while the PE fraction contained much less AA than the FGE-salmon-PLs, implying that the AA contained in the FGE-salmon-PLs is distributed in PLs subclasses other than the PC and PE species.

The ω6/ω3 ratio seems to be a determinant of the platelet adhesion, since supplementation of fish oil rich in ω3 PUFA with an ω6/ω3 ratio of 0.1 resulted in a reduction of human platelet adhesiveness stimulated by thrombin or adenosine diphosphate (ADP), whereas supplementation of soy lecithin with an ω6/ω3 ratio of 3.0 resulted in a stimulatory effect on resting and stimulated platelet adhesion [49]. In the present study, the ω3 fatty acid content of the PC and PE fractions was approximately similar to that of ω6 fatty acids, and thus the ratio of ω6/ω3 PUFA is similar to the value of 1, which is much lower than that of Westernised diets and is within the range of 5/1–20/1 [50]. It has been reported that high values of this ratio are correlated with a higher risk in cardiovascular disease and other chronic diseases [50]. Taking this into account, the observed favorable ω6/ω3 ratio in the bioactive PC and PE fractions of the FGE-salmon-PLs extract may also provide further evidence for their potential cardioprotective properties, since the lower this ratio is in the diet, the better health outcomes it provides against such chronic diseases [50]. In addition, the presence of odd-chain fatty acids, such as pentadecanoic acid (15:0) and heptadecanoic acid (17:0) in the bioactive PC and PE fractions of the FGE-salmon-PLs extract seems to add to the cardioprotective properties to this extract, since the presence of such diet-derived odd-chain fatty acids in human plasma PL has been associated with a decreased risk for CVD and type II diabetes and with favourable effects against the atherogenic leptin and plasminogen activator inhibitor-1 (PAI-1) levels [51,52,53,54].

Moreover, our LC-MS data also shows that the bioactive PC and PE lipid subclasses are mostly composed of diacyl-PC and diacyl-PE species containing 14–22 carbon chains with 0, 1, or 2 double bonds at the *sn*-1 position and ω3 PUFA (mostly either DHA or EPA) or MUFA (such as oleic acid) at the *sn*-2 position, while 1-*O*-alkyl-2-*sn*-acyl-PC moieties also seem to be present in less but considerable quantities, usually with ω3 PUFA (mostly either DHA or EPA) also at the *sn*-2 position, see Figure 3, Figure 4 and Figure 6. Our results are in accordance with previously reported research concerning the compositional analysis and positional distribution of fatty acids (mostly at the *sn*-2 position) in phospholipids extracted from different samples of fillets of Atlantic salmon (*Salmo salar*) that were fed different diets (i.e., Vegetable and Fish Oil Blends) [44], but also in phospholipids of several other fish, marine mammals, and krill oil [45,46,47,55].

The results of this study are partly in accordance with the previously reported structural elucidation of the bioactive PC lipid subclass of the CE-salmon-PLs [16], since apart from differences in the bioactivities, some structural differences were also observed. These structural differences are due to the high abundance of PE moieties (either diacyl-PE or alkyl-acyl-PE) baring EPA and DHA at their *sn*-2 position within the most bioactive TLC-derived lipid fraction corresponding to the PE lipid subclass of the FGE-salmon-PL. These results were not previously observed in the relative PE lipid subclass of the CE-salmon-PL [16].

In another study [42], different extraction protocols (i.e., a chloroform-free solvent extraction protocol based on 2-methoxy-2-methylpropane) resulted in similar phospholipid composition and distribution of fatty acids in seabass brains derived from the well-established conventional method of Bligh and Dyer [20]. However, it should be noted that the solvents used in the aforementioned study were different than those used in the present study and they were also not of food-grade standard.

Similarly to the present study, using different solvents and extractions (either food-grade or conventional) in other marine sources (i.e., seaweed or microalgae) resulted in differences in the lipid yield [43,56] and the fatty acid composition (i.e., an EtOH-based food-grade extraction achieved an extract rich in PUFA in contrast to conventional methods) [43]. Furthermore, polar lipid fractions, rich in phospho- and sphingolipids, obtained from another food source (buttermilk) by using food-grade ethanol gave a higher yield than those obtained using non-food-grade solvents, while these food-grade extracted PL fractions exhibited strong antiproliferative bioactivities against several cancer cells, whereas the non-food-grade extracted lipid extracts and fractions did not provide any significant anti-proliferative activity in any of the tumour cell lines that were assessed [57]. Therefore, according to our results and to the relative results of the aforementioned studies, it is suggested that the observed differences in PC and PE compositions and bioactivities in this study can be attributed to the use of food-grade extraction solvents and methods.

Concerning the presence of alkyl-acyl-moieties present in both the PC and PE fractions of the FGE-salmon-PLs, specific alkyl-acyl-PC and alkyl-acyl-PE baring DHA at the *sn*-2 position also appears to be present in the TLC fractions of the bioactive PC and PE lipid subclasses of the FGE-salmon-PLs, namely 1-*O*-alkyl-(18:0)-2-*sn*-alkyl-(22:6,DHA)-3-PC and 1-*O*-alkyl-(16:0)-2-*sn*-alkyl-(22:6,DHA)-3-PE, respectively, which were also reported to be present in the relevant TLC fractions of the bioactive PC and PE lipid subclasses of the CE-salmon-PLs [16]. Interestingly, other alkyl-acyl-PC and alkyl-acyl-PE baring EPA at the *sn*-2 position seem also to be present in the TLC fractions of the bioactive PC and PE lipid subclasses of the FGE-salmon-PLs, such as 1-*O*-alkyl-(20:0)-2-*sn*-alkyl-(20:5,EPA)-3-PC and the 1-O-alkyl-(20:0)-2-sn-alkyl-(20:5,EPA)-3-PE, which were not found in the bioactive PC and PE lipid subclasses of the CE-salmon-PLs [16].

Ether phospholipids (i.e., alkyl-acyl PLs) are usually found in animal tissues and human cells as minor components, existing together with molecular species of diacyl phospholipids carrying the same polar head group [58]. Dietary 1-*O*-alkyl-*sn*-2-DHA phospholipid species may easily cross through the intestinal barrier because the rate of hydrolysing *sn*-1-ether fatty chains/*sn*-2-DHA phospholipids by phospholipases can be significantly slowed down [59,60], and most of the species are relatively stable in the metabolism with high-density lipoprotein (HDL) in vivo, compared with *sn*-1-acyl fatty chains/*sn*-2-DHA phospholipid species. In addition, such ether phospholipid species, are more stable in in vivo lipid metabolism, compared with related acyl species, since they can survive from blocking due to the hydrolysis of phospholipase A_1_ and phospholipase A_1_-like enzymes in vivo metabolism [61,62,63]. Thus, ω3 PUFA containing ether phospholipid species can be delivered smoothly and unaffected by lipid metabolism, into plasma lipoproteins and from there to several blood cells and tissues, including those of difficult accessibility such as the brain.

After being transferred to blood cells such as platelets, alkyl-acyl-phospholipids either possess a strong inhibitory or a weak agonistic effect or both effects (in different concentrations) against the PAF pathways of activating cells (including platelet aggregation), because of their structural resemblance to the PAF molecule, and thus their antagonistic effect for its receptor [5]. Similarly to the previously described results for the CE-salmon-PLs [16], in the present study, there were no platelet aggregatory agonistic effects against PAF in all of the PL samples and PL lipid subclasses that were tested. It seems that the FGE-salmon-PLs possess stronger inhibitory effects against the PAF pathway than any possible agonistic effect from the constituent lipids in the PL fractions, and in this case, these components are practically acting mostly as PAF inhibitors.

In general, membrane phospholipids are essential in blood coagulation reactions. The cell membrane, which is characterised as the “main location” of blood coagulation, is one of its regulatory factors, and changes in PC and PE content and the phospholipid composition of the cell membrane regulate the coagulation reactions [64] and the relative responses of the bindings of agonists such as PAF and thrombin to their receptors. The process of fibrinogen conversion into fibrin and thus the formation and morphology of fibrin clot is also affected by the charge and phase state of lipids, especially polar lipids, in membrane surfaces [65]. Thus, coagulation and anti-coagulatory reactions are coordinated and controlled by changes in the phospholipid composition of the cellular membrane where the coagulation reaction takes place [64]. In addition, exposure of platelets to thrombin usually induces a reduction of the amounts of specific phospholipid subclasses, including PC and PE, in the plasma membrane of thrombin-stimulated platelets [66], while PC and PE have been found to suppress the rate of thrombin formation and blood coagulation [64]. PE is also physically present at the luminal endothelial surface, where it tentatively functions as a critical anticoagulant [67], while PE rich in PUFA enhance the anticoagulant activity of thrombomodulin in endothelial cells [68].

Remarkably, PC and PE rich in EPA also reduce thrombin-induced inflammatory and atherosclerotic effects [69]. For example, when endothelial cells were cultured in the presence of EPA incorporated in PC and PE, a significant reduction of the thrombin-induced intracellular release of AA metabolites and the thrombin-evoked release of endothelin-1 was observed, which is a vasoactive compound implicated in hypertension, atherosclerosis, and CVD [69]. In addition, supplementation of cod liver oil rich in EPA in healthy human subjects for at least 14 days resulted in a reduction of thrombin-induced platelet aggregation and alteration of the FA composition (by incorporating EPA and DHA) in all PLs subclasses of human platelets [70]. Thus, our results indicating the presence of specific diacyl- or alkyl-acyl- subspecies of bioactive PC and PE moieties carrying EPA at the *sn*-2 position, seems to explain the higher potency of this novel FGE against the thrombin pathways, in contrast to the relative PC and PE subclasses of the CE-salmon-PL.

Furthermore, a dioleoyl-PE molecule was also present at the PE fraction (with *m/z* 743.5 and retention time 2.554 min), while liposomes containing such PE and PC molecules also promote heparin’s anticoagulant effect against the thrombin-induced coagulation time, among other agonists [71]. 

Apart from the bioactivities of the PC and PE fractions, we have also found that the SM family fraction of the FGE-salmon-PLs exhibited higher anti-PAF and anti-thrombin activities in comparison to the reported anti-PAF and anti-thrombin activities of the fraction of the SM family of the CE-salmon-PLs [16]. The anti-thrombin effects of the fraction of the SM family of the FGE-salmon-PLs were found to be similar to the relative anti-thrombin effects of the PC fraction and lower to that of the PE fraction of the FGE-salmon-PLs. In addition, the anti-PAF effects of the fraction of the SM family of the FGE-salmon-PLs was found to be slightly lower (but not significantly lower, *p* > 0.05) than the relative effect of the PC fraction, whereas it was significantly lower (*p* < 0.05) than the relative effect of the PE fraction of the FGE-salmon-PLs. Furthermore, within the TLC fractions of PC and PE, the LC-MS analysis revealed that only PC and PE polar lipids exist, whereas in the TLC fraction of SM, there are usually several other classes of bioactive polar lipids belonging to the SM family that co-migrate (such as sphingomyelin, cerebrosides, ceramides, gangliosides and several other glyco-sphingolipids). Moreover, several lipid molecules belonging to the SM family mainly affect thrombin generation [72]. Therefore, more studies are required to elucidate all the species of lipids of the SM family and their effects on the PAF and thrombin pathways.

The fact that such food and especially, marine-derived bioactive PLs (i.e., PC and PE baring EPA, DHA and oleic acid at their *sn*-2 position, and with low ω-6/ω-3 ratio, but also several lipids of the SM family) can be incorporated with high bioavailability and act synergistically in blood lipoproteins and membranes of circulating blood cells and endothelial cells [1,2,3,4], seems to be related to their abilities to beneficially alter the functionality of such key cells and pathways of inflammation and coagulation [5]. For example, the addition of farmed Atlantic salmon to the diet twice a week for four weeks at portions of 180 g and 270 g modified plasma phospholipid fatty acid (PLFA) proportions of ω3 and ω6 in a level associated with decreased risk for CVD [73]. In addition, supplementation of marine PLs rich in ω3 PUFA in prostate cancer patients resulted in a favourable increase of EPA and DHA in blood lipids, while AA (ω6 PUFA) decreased significantly, suggesting a mechanism for a lower incidence of metastatic progression in prostate cancer patients with high consumption of fish containing PLs rich in ω3 PUFA [74].

Taking into account all the above, in combination with the strong antithrombotic effects of the FGE-salmon-PLs extract against both PAF and thrombin pathways, one may suggest that this extract may be a promising candidate for the development of novel cardioprotective food supplements and nutraceuticals. In addition, such extracts rich in bioactive PLs baring ω3 PUFA can also be used effectively in other inflammation-related disorders [5]. Of interest are the disorders of the central nervous system, since such bioactive lipids can more effectively reach and surpass the blood–brain barrier [5]. 

However, ex vivo and in vivo studies are required to further support such a notion. Nevertheless, the fact that these FGE-salmon-PL extracts were derived from a sustainable salmon source, with the use of food-grade solvents is a promising supportive element towards its future use as a food supplement and nutraceutical, to be tested in clinical trials in several inflammation-related disorders, including CVD.

## 4. Materials and Methods

### 4.1. Materials and Instrumentation

All glass and plastic consumables, reagents, and solvents were of analytical grade and were purchased from Fisher Scientific Ltd. (Dublin, Ireland). Evacuated sodium citrate S-monovettes and 20G safety needles for blood sampling were purchased from Sarstedt Ltd (Wexford, Ireland). The preparative TLC glass plates (20 × 20 cm) with silica gel G-60 and 1.0/2.0 mm thickness were purchased from Merck (Darmstadt, Germany). The platelet aggregation bioassay was carried out on a Chronolog-490 two-channel turbidimetric platelet aggregometer (Havertown, PA, USA), coupled to the accompanying AGGRO/LINK software package. All platelet aggregation consumables were purchased from Labmedics LLP (Abingdon on Thames, UK). Standard PAF, thrombin, egg phospholipid extract, and BSA were purchased from Sigma Aldrich (Wicklow, Ireland). Centrifugations were carried out on an Eppendorf 5702R centrifuge (Eppendorf Ltd., Stevenage, UK). Spectrophotometric analysis was carried out on a Shimadzu UV-1800 spectrophotometer (Kyoto, Japan). 

### 4.2. Salmon Samples Assessed

A sustainable marine source was chosen for this study; Irish organic farmed salmon (*Salmo salar*). All salmon fillets were provided by the same supplier, Marine Harvest (Co. Donegal, Ireland). More specifically, the Irish organic farmed salmon used in this study was produced at the Marine Harvest farming facilities with a diet containing only organic approved natural ingredients from sustainable sources, with fish meal and oil derived from the trimmings of fish caught for human consumption. All ingredients were free from genetically modified organisms. Fish were reared in large pens which allowed them to follow their natural shoaling behaviour. Pens contained less than 10 kg/m^3^, which is less than half that of conventional farms. The production sites were continuously flushed with clean water, preventing any build-up of parasites or pollutants. This natural, healthy environment and low population density allowed the fish to develop good muscle tone and body shape. All salmon fillets tested were harvested at the same time (same lot number) within the Marin Harvest farming facilities. Therefore, for the comparison between the CE-salmon-PLs and FGE-Salmon-PLs, all analyses on all of the extracts were conducted on salmon fillets from the same batch of farmed salmon. 

### 4.3. Isolation of FGE-Salmon-PLs from Salmon Fillets

Several (*n* = 6) 100 g samples of the fresh salmon fillets were homogenised mechanically by a Waring blender (Fisher Scientific Ltd, Dublin, Ireland) and their total lipids (TL) were extracted and further separated into their neutral lipids (NL) fraction and the polar lipids fraction (FGE salmon-PLs) using food-grade solvents, namely water, ethanol, and hexane (all of HPLC grade), according to EU legislation for food-grade based extractions of fish oil (consolidated Directive 2009/32/EC: https://eur-lex.europa.eu/legal-content/en/ALL/?uri=CELEX:32009L0032). Solvents were evaporated from the samples using flash rotary evaporation (Buchi Rotavapor, Mason Technology, Dublin, Ireland) and lipid samples were transferred into small glass vials, where all the remaining solvents were further evaporated under a stream of nitrogen. The acquired FGE-salmon-PL extracts were weighed and stored under a nitrogen atmosphere in −20 °C for further analysis.

### 4.4. Fractionation of FGE-Salmon-PLs to Subclasses by Preparative TLC

The TLC analysis of the FGE-salmon-PLs was performed as previously described [16]. Briefly, up to 50 mg of TPL was applied to the TLC plates. An elution system consisting of chloroform:methanol:water 65:35:6 (*v/v/v*), was utilised for the separation of FGE-salmon-PLs. Subsequently, the plates were stained under iodine vapours. Six major bands appeared after the separation of the FGE-salmon-PLs. Following the evaporation of the iodine vapours, the bands were scraped, and lipids were extracted from the silica gel according to the Bligh and Dyer method [20]. The chloroform phase was evaporated to dryness under nitrogen and lipids were weighed, re-dissolved in 1 mL of chloroform:methanol 1:1 (*v/v*), and stored at −20 °C in a nitrogen atmosphere until further analysis.

### 4.5. Human Platelet-Rich Plasma (hPRP) Aggregation Studies of FGE-Salmon-PLs

All experiments were conducted as previously described [16]. Briefly, for hPRP isolation, healthy human volunteers (*n* = 10) donated fasting blood samples. The Ethics Committee of the University of Limerick approved the protocol and it was performed in accordance with the Declaration of Helsinki. Healthy donors were fully aware that their blood samples were used in our study and written consent was provided to the specialised phlebotomist. The blood samples were in sodium citrate anticoagulant and were centrifuged at 194 g for 18 min at 24 °C with no brake applied. The supernatant hPRP was then transferred to polypropylene tubes at room temperature for the aggregation bioassays, whereas platelet-poor plasma (PPP) was obtained by further centrifuging the specimens at 1465 g for 20 min at 24 °C with no brake applied. hPRP was adjusted to 500,000 platelets/µL if required by addition of the respective volume of PPP according to the absorbance of the hPRP measured in spectrophotometer. 

Aliquots of standard PAF solution in chloroform/methanol (1:1 *v/v*) were evaporated under a stream of nitrogen and re-dissolved in bovine serum albumin (BSA; 2.5 mg BSA/mL saline) into cuvettes to obtain PAF solutions with final concentrations ranging from 2.6 × 10^−8^ to 2.6 × 10^−5^ mol/L. The examined salmon PL samples were also dissolved in BSA (2.5 mg BSA/mL saline). Standard active thrombin was dissolved in saline prior to testing. The maximum reversible PAF-induced/thrombin-induced platelet aggregation was determined as 100% aggregation, that was also used as baseline (0% inhibition), by adding PAF at approximately 2.6 × 10^−8^ M final concentration or thrombin at approximately 0.01–0.4 U/mL in the aggregometer cuvette. The PAF-induced/thrombin-induced aggregation was calculated first at 0% inhibition of baseline in a cuvette, whereas after the pre-incubation of hPRP with the test samples in a variety of concentrations in a different cuvette, the same amount of PAF/thrombin was added, and the reduced aggregation was calculated. Thus, a linear curve at the 20–80% range of the percentage of inhibition against PAF-induced/thrombin-induced aggregation of hPRP to the concentrations of each sample was deduced. From this curve, the concentration of the sample that led to 50% of PAF-induced/thrombin-induced aggregation of hPRP was calculated as the 50% inhibitory concentration value, also known as the IC_50_ value for each sample. All experiments were performed in triplicate (*n* = 3), using a different donors blood sample for each replicate, to ensure reproducibility. The resulting IC_50_ values were expressed as a mean value of the mass of lipid (µg) in the aggregometer cuvette ± standard deviation (SD).

### 4.6. LC-MS Analysis of FGE-Salmon-PLs

FGE-salmon-PLs and the most bioactive TLC-derived lipid fractions (corresponding to the PC and PE lipid subclasses) against both the PAF pathway and the thrombin pathway, and the FFAs that were derived from their saponification, were further analysed by LC-MS as previously described [16].

Briefly, each of these lipid samples was separated into two half parts and dried in a N_2_ stream. The first half of each sample was saponified by adding 1.5 mL of saponification reagent, (2.5 M KOH: methanol (1:4, *v/v*)), which was gently vortexed. The vials were incubated at 72 °C for 15 min prior to the addition of 225 µL of formic acid. Then, 1725 µL of chloroform and 375 µL of Milli-Q water were added, and vortexed to separate the two layers. The chloroform layer containing free fatty acids was transferred carefully to amber vials and evaporated to dryness before being stored at −20 °C until LC-MS analysis.

Before LC-MS analysis, all of the dried lipids were re-constituted in 500 µL of methanol: dichloromethane (2:1, *v/v*), centrifuged at 13,793 g for 6 min (Heraeus Biofuge Stratos, Fisher Scientific Ltd., Dublin, Ireland) prior to filtering through 3 kDa ultra-centrifuge filters (Amicon Ultra 3k, Merck Millipore Ltd., Carrigtwohill, Co. Cork, Ireland). Polar lipid and free fatty acid profiles were obtained in a HPLC (Agilent 1260 series, Agilent Technologies Ireland Ltd., Little Island, Co. Cork, Ireland) equipped with a Q-TOF mass spectrometer (Agilent 6520) and the source type was electrospray ionization (ESI). The column used for separations was an Agilent C18 Poroshell 120 column (2.7 µm, 3.0 × 150 mm). The composition of the mobile phase (A) was 2 mM ammonium acetate in water and 2 mM ammonium acetate in 95% acetonitrile for the mobile phase (B). Chromatographic separation was performed by gradient elution starting with 60% B for 1 min, then increasing to 90% B over 2.5 min. Subsequently, 90% B was held for 1.5 min and increased afterwards to 100% over 5 min. Then, 100% B was held for 4 min, reducing afterwards to 60% B over 0.5 min and held for 1 min until the next run. The mobile phase flow rate was 0.3 mL/min until 5 min elapsed, increasing up to 0.6 mL/min after 10 min and held at this flow rate until the end of the run. The injection volume was 10 μL. The mass spectrometer was operated in negative ionization mode, scanning the lipids from *m/z* 50–1100. Drying gas flow rate, temperature, and nebuliser pressure were at 5 L min^−1^, 325 °C, and 30 psi, respectively. Fragmentor and skimmer voltages were kept at 175 V and 65 V, respectively, and the capillary voltage was 3500 V. In the negative ion mode, the monitoring reference masses used were 1033.988 and 112.9855, respectively.

The assignment of free fatty acids and phospholipid species was based upon a combination of survey, daughter, precursor, and neutral loss scans. The identity of phospholipid species was verified using the LIPID MAPS: Nature Lipidomics Gateway (www.lipidmaps.org), by using the lowest delta values combined with the results obtained from the LC-MS analysis of the FFA that were produced by their saponification.

### 4.7. Statistical Analysis

One-way analysis of variance (ANOVA) was used in order to find the significant differences between IC_50_ values against PAF- and thrombin-induced platelet aggregation of all the samples tested, but also when these values were compared with previously reported ones for the CE-salmon-PLs [19]. Differences were considered to be statistically significant when the *p*-value was less than 0.05. The data were analysed using a statistical software package (IBM-SPSS statistics 24 for Windows, SPSS Inc., Chicago, IL, USA).

## 5. Conclusions

We have previously described that several marine sources, including salmon, contain bioactive PLs with strong antithrombotic and anti-atherogenic cardioprotective activities [4,13,14,15,16,18,19,22]. However, such PL extracts were previously obtained from these sources by using conventional extractions with non-food-grade solvents such as chloroform and petroleum ether [20,21]. Thus, such CE-salmon-PLs extracts cannot be used in human-based trials and dietary interventions or for developing novel food supplements and nutraceuticals.

To the best of our knowledge, this is the first study to report that PLs derived from salmon using food-grade solvents in compliance with current EU legislations exhibited strong antithrombotic activities against both the PAF and thrombin pathways of platelet aggregation. Remarkably, the FGE-salmon-PLs extract, as well as each one of its most bioactive lipid subclasses (PC and PE), exhibited much higher anti-thrombin effects than the relative CE-salmon-PLs extract. Moreover, our LC-MS-based structure–activity relationship studies revealed that the existence of not only DHA but also EPA at the *sn*-2 position of specific subspecies of bioactive diacyl- or alkyl-acyl- PC and PE moieties, seem to be related to the more potent anti-thrombin activities observed in the FGE-salmon-PLs, in contrast to the anti-thrombin activities of CE-salmon-PLs. 

Taking into account that both thrombin and PAF are potent mediators implicated in inflammatory manifestations related to endothelial dysfunction and the genesis and progression of atherosclerosis and subsequent cardiovascular disorders [5], our results indicate that the FGE-salmon-PLs are strong candidates for the development of cardioprotective supplements and nutraceuticals. However, pre-clinical/clinical studies are required in order to acquire evidence for the in vivo beneficial effects of such FGE-salmon-PLs-based food supplements and nutraceuticals.

## Figures and Tables

**Table 1:** Yield of FGE-salmon-PLs of Irish organic farmed salmon (*Salmo salar*) fillets, and the inhibitory effect of these lipids towards the PAF and thrombin pathways of platelet aggregation in hPRP, in comparison with the previously reported data of the CE-salmon-PLs. Reproduced with permission from Tsoupras et al. [16].

Yield & Bioactivity of Salmon-PLs	CE-Salmon-PLs ^&^	FGE-Salmon-PLs
Yield of PLs ^$^ (g) ± SD	0.86 ± 0.36	0.61 ± 0.21
IC_50_^†^ (µg) ± SD against PAF–induced platelet aggregation	45 ± 22	86 ± 18 *
IC_50_^†^ (µg) ± SD against thrombin-induced platelet aggregation	382 ± 39	102 ± 29 *

^$^ Expressed as mean values of g of lipids per 100 g of salmon fillet (mean ± SD, *n* = 6); ^†^ IC_50_ values reflect the inhibitory strength of each PL extract towards PAF/thrombin-induced platelet aggregation in hPRP and is expressed as mean values of μg of lipids in the aggregometer cuvette that cause 50% inhibition on PAF/thrombin-induced platelets aggregation in hPRP ± standard deviation; ^&^ the yield of extraction and the IC_50_ values of the CE-salmon-PLs against PAF and thrombin-induced aggregation of hPRP are reproduced in this table (2nd column) as reported by Tsoupras, Lordan, Demuru, Shiels, Saha, Nasopoulou, and Zabetakis [16], in order to facilitate comparisons between the newly acquired data of the present study (3rd column). * Statistical significant difference (*p* < 0.05) when compared with the previously reported IC_50_ values of the CE-salmon-PLs extracts [16].
